# Liver Abscess Due to an Unusual Foreign Body: A Case Report

**DOI:** 10.7759/cureus.87420

**Published:** 2025-07-07

**Authors:** Salaheddine Abdennebi, Amina Babana El Alaoui, Lahcen Ifrine, Abdelkader Belkouchi, Omar Belkouchi

**Affiliations:** 1 General Surgery, Ibn Sina Hospital, Faculty of Medicine and Pharmacy of Rabat, Mohammed V University, Rabat, MAR; 2 Surgery, Faculty of Medicine and Pharmacy of Rabat, Mohammed V University, Rabat, MAR; 3 Surgery A, Moulay Abdellah Hospital, Salé, MAR

**Keywords:** fishbone, foreign body, laparoscopy, minimally invasive liver surgery, pyogenic liver abscess

## Abstract

Pyogenic liver abscess (PLA) is an uncommon and potentially threatening condition. However, foreign body-induced PLA due to gastrointestinal perforation is very rare and challenging to diagnose, especially when there is no recalled history of ingestion and no signs of peritoneal irritation. Its management is still not standardised and can be very difficult, leading, in almost all cases, to surgery. We present the case of a 60-year-old patient with a PLA resistant to medical treatment. Further investigation revealed a radio-dense image in the left hemiliver. The patient underwent laparoscopic surgery and removal of a foreign object deemed to be a fishbone.

## Introduction

Pyogenic liver abscess (PLA) is defined as a collection of pus composed of inflammatory cells, mainly neutrophils, and cellular debris due to infection, which induces necrosis of the surrounding liver tissue. Causes may include infection of bile ducts, hematogenous and contiguous contamination from the abdomen, or trauma [1]. Ingestion of foreign bodies (FBs) is a common complaint in the emergency department, although most cases resolve uneventfully. It can lead to serious complications such as perforation of the gastrointestinal tract, inducing intraperitoneal abscesses [2]. Liver abscesses induced by the migration of an FB through the digestive wall are very rare and mostly overlooked, as patients do not recall the ingestion [3]. Misdiagnosis as cryptogenic liver abscess is frequent without proper imaging, and outcomes can be lethal without the removal of the FB [4]. The purpose of this case report is to emphasize the difficulties encountered in the management of this rare condition.

## Case presentation

**Figure 1:**
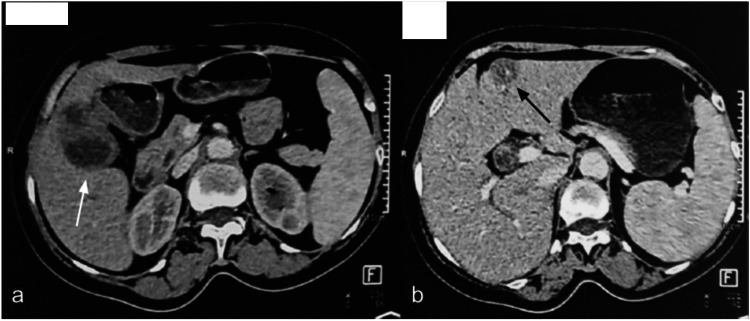
First abdominal contrast-enhanced computed tomography scan showing two liver abscesses. (a) Abscess lateral to the gallbladder (white arrow); (b) Abscess in the left lobe (black arrow). No underlying cause was identified (appendicitis, cholecystitis, tumor, foreign body).

A 60-year-old male patient with no medical history, notably no use of alcohol or tobacco, was admitted to the emergency department due to the manifestation of fever and abdominal pain. Diagnosis of hepatic abscess was made based on computed tomography (CT) findings (Figure 1), and the patient was treated using antibiotic therapy.

After an initial favorable response, the patient presented again with fever 15 days into treatment. He sought care at another facility, where an abdominal ultrasound revealed regression of the abscess located near the gallbladder and persistence of the abscess in the left lobe. An ultrasound-guided puncture was performed, which yielded purulent fluid with no identifiable pathogens.

**Figure 2:**
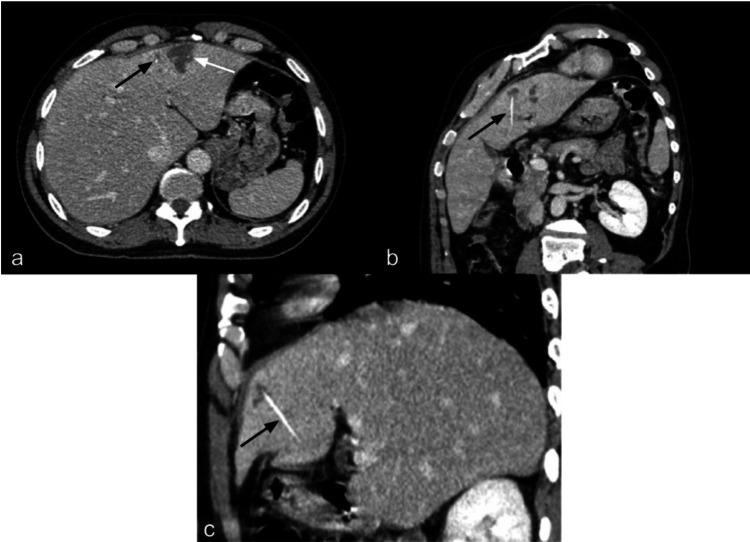
Second abdominal contrast-enhanced computed tomography scan showing a relapse of the left lobe abscess. (a) Transversal view showing a left lobe abscess (white arrow) and a radiodense structure (black arrow); (b) Coronal view showing a radiodense linear structure (black arrow); (c) Sagittal view showing a radiodense linear structure (black arrow).

The patient received empirical antibiotic therapy for six weeks. Follow-up CT scan imaging revealed the persistence of the left lobe abscess; however, a radiodense image was observed that was suggestive of an intrahepatic FB (Figure 2). The patient denied any recent ingestion of FBs. He was referred to our department for surgical management.

Upon admission, the patient was stable, apyrexial, and physical examination revealed mild tenderness in the right upper quadrant with no signs of sepsis. Biological tests were normal, with a normal C-reactive protein (CRP) level and white blood cell count (Table 1).

**Table 1:** Preoperative biological analysis. WBC: white blood cells; PT: prothrombin time; CRP: C-reactive protein; ASAT: aspartate aminotransferase; ALAT: alanine aminotransferase; GGT: gamma-glutamyl transferase

Analysis	Patient value	Reference value	Unit
Hemoglobin	14	12.1 - 16.6	g/dl
WBC Count	7900	3.8 - 11.0	/mm³
Platelets	237000	150,000 - 450,000	/mm³
PT	93%	> 70	%
CRP	4	< 6	mg/l
ASAT	32	5 - 34	UI/L
ALAT	39	0 - 55	UI/L
Total Bilirubin	7	2 - 12	mg/l
GGT	57	11 - 59	UI/L

**Figure 3:**
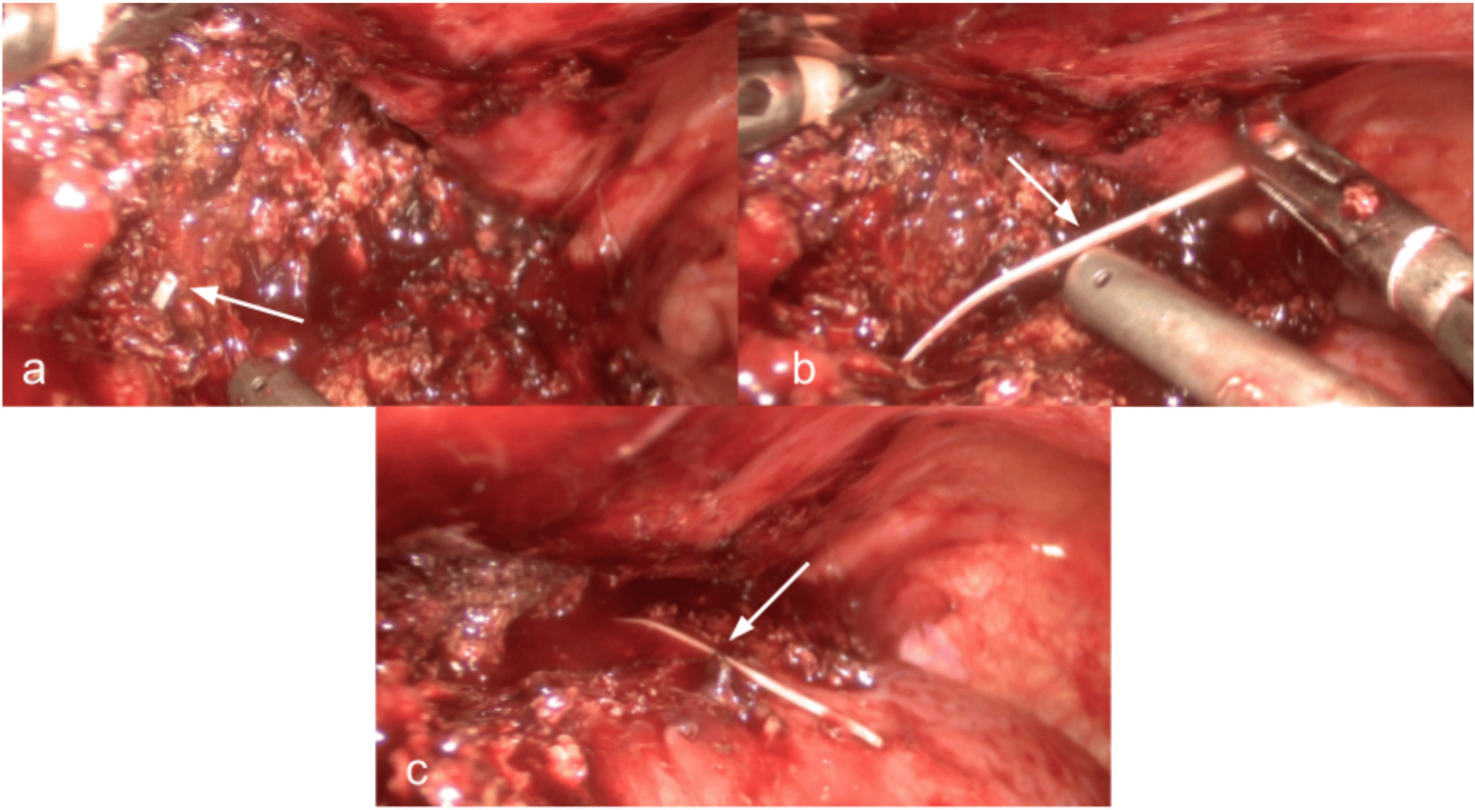
Laparoscopic intraoperative view showing liver parenchymal transection, and extraction of the foreign body. (a) Tip of the foreign body (white arrow) after transection; (b and c) Extraction of the foreign body, approximately 5 cm long.

**Figure 4:**
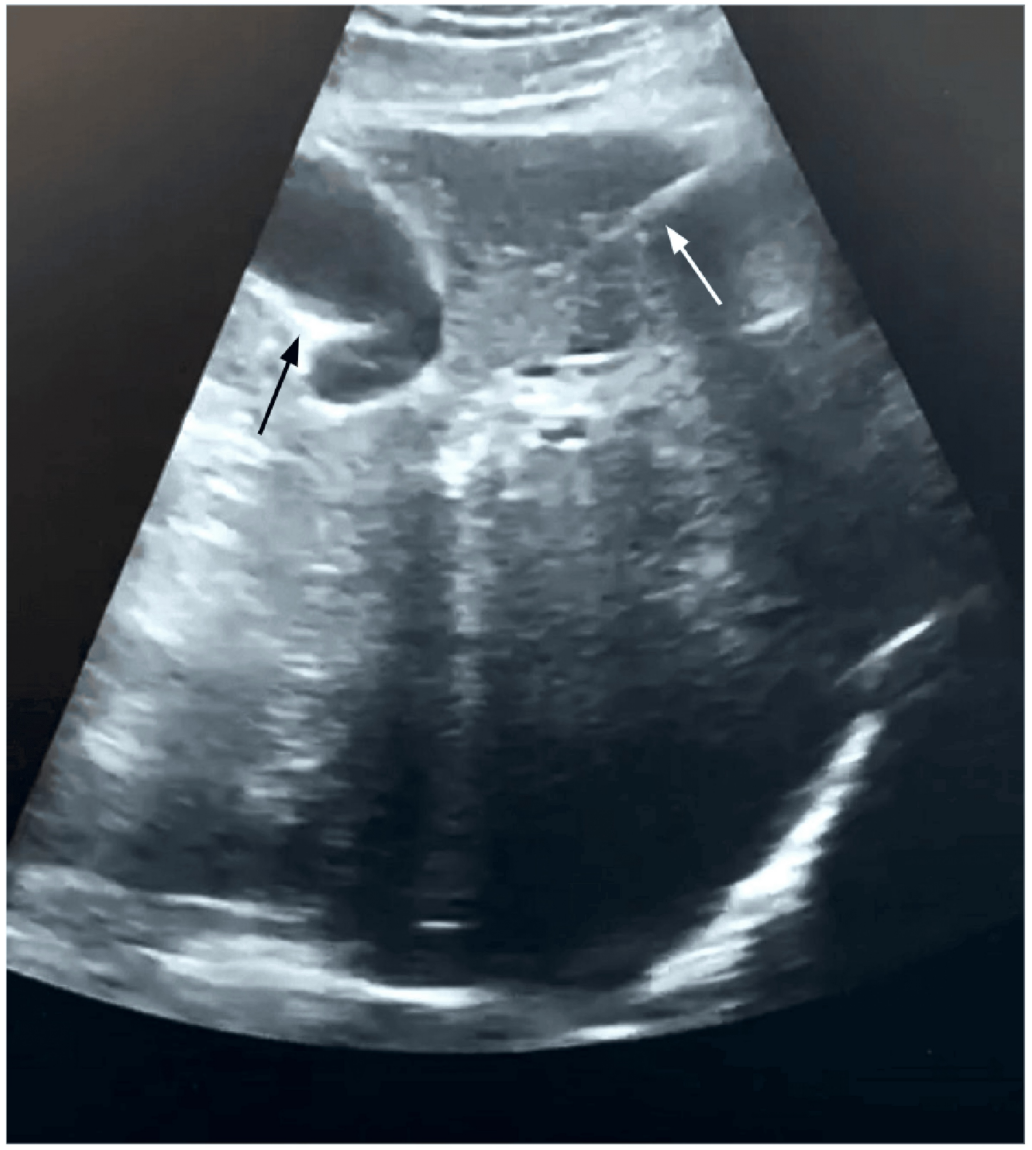
Intraoperative ultrasound showing the foreign body (white arrow) and the gall bladder (black arrow).

**Figure 5:**
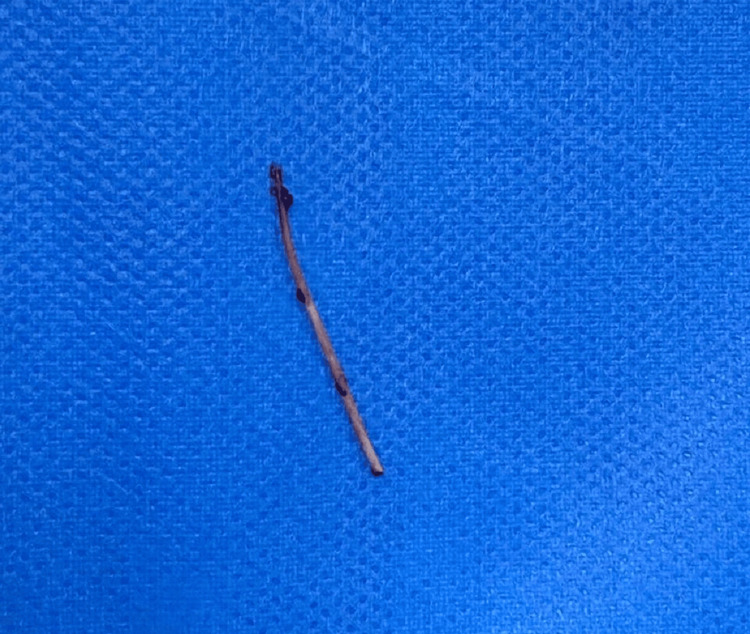
Foreign body identified as a fishbone.

The FB was extracted by laparoscopy using three ports (Figure 3), and under intraoperative ultrasound (Figure 4). The object was identified as a fishbone (Figure 5).

The patient was discharged on postoperative day one. The postoperative follow-up was satisfactory at one month, with normal physical and ultrasound examination. The subsequent work-up did not reveal any issues, and the patient was referred to his hepatologist.

## Discussion

In most cases (80-90%), ingested FBs pass uneventfully through the gastrointestinal tract and are eliminated in feces within a week [2,3,5-7]. Only 1% result in perforation, which occurs in the ileo-caecal and recto-sigmoïdal regions of the lesser gastrointestinal tract and causes intraperitoneal abscesses with acute symptoms [2,3,8].

The first case of PLA due to migration of a perforating FB was described by Lambert in 1898 [9]. It is exceptional with an incidence of less than 1%, and can often be misdiagnosed with cryptogenic liver abscesses given that the history of ingestion is often unknown to patients with low oral sensations, especially in the elderly and children, and do not exhibit any signs of gastrointestinal perforation [3,6,8,9]. Symptoms are absent for a long period, and they are nonspecific when apparent. Patients report upper abdominal pain in 70-85% of cases, and general symptoms such as fever and chills (50-70%) without underlying conditions, which contribute to the misdiagnosis. Patients can be admitted with sepsis or septicemia resistant to antibiotics in severe cases [5,10-12]. Laboratory investigation results are also nonspecific, with elevated WBC and CRP suggesting inflammation and infection. Liver markers are within normal range or slightly elevated [11,12].

As for the implicated pathogens, a single strain of bacteria was found in most cases, around 54%, two bacterial flora and multi-flora around 18% and 13%, respectively, while cultures were negative in 14.5% of cases. Amongst all identified bacteria, *Streptococcus* species were the most common (72.3%), followed by *Escherichia coli* (17%) and *Klebsiella* (10%). These findings corroborate the gastrointestinal origin of the infection, the FB acting as a vector for the pathogens [5,13,14].

Ultrasonography can easily detect the abscess and its location, which in most cases, is the left lobe of the liver, given that the migration of the FB involves a perforation of the gastric antrum, pylorus, or the first and second part of the duodenum. Cases of right hepatic lobe injuries have been reported, with the migration being processed through the duodenum or the right colon [2,8,10,15]. However, the FB can be unnoticed, especially when its ingestion is not reported in the patient's history, and when it is small, thin, and poorly echogenic, such as fish bones [12]. Therefore, abdominal contrast-enhanced CT is considered to be highly reliable in identifying the FB and its nature with a sensitivity of 100% and specificity of 90%; however, it can sometimes be misinterpreted as surgical clips or artifacts [3,5,10].

The management of PLA due to FB migration typically involves empirical broad-spectrum antimicrobial treatment prior to imaging-guided percutaneous drainage, enabling the identification of the pathogen in the pus if previous blood cultures are not successful, followed by minimally invasive procedures to remove the FB. Studies suggest that the cure rate is very low in patients treated with antibodies and drainage alone compared to those who underwent minimally invasive procedures; recurrence and hospital stay were also high [2,5,6,8,10-12].

Ultrasonic endoscopy can be performed only in some early cases when the gastrointestinal mucosa is not completely healed, enabling the identification of the tip of the FB. Therefore, the surgical approach is considered crucial in the removal of the FB and is typically performed by a three-port laparoscopy, given that most cases are reported in a late stage when the FB is already inside the liver and no gastrointestinal perforation was observed [2,3,5,12,14].

## Conclusions

PLA caused by the migration of an ingested FB through the gastrointestinal tract is rare but challenging to diagnose and treat. This case shows the importance of considering FB-induced liver abscess in patients with cryptogenic abscesses, especially when there is no response to conventional treatment. The lack of a clear history of FB ingestion should not exclude this diagnosis, given that patients do not often recall such events. The role of high-resolution imaging modalities, such as contrast-enhanced CT, is critical in the identification of occult intrahepatic FBs. Surgical removal, particularly by mini invasive procedures, remains the cornerstone of managing this entity, significantly reducing the recurrence rate and complications.
